# Identification of *Wolbachia* Strains in Mosquito Disease Vectors

**DOI:** 10.1371/journal.pone.0049922

**Published:** 2012-11-21

**Authors:** Jewelna Osei-Poku, Calvin Han, Charles M. Mbogo, Francis M. Jiggins

**Affiliations:** 1 Department of Genetics, University of Cambridge, Cambridge, United Kingdom; 2 Centre for Geographic Medicine Research, Kenya Medical Research Institute, Kilifi, Kenya; University of Poitiers, France

## Abstract

*Wolbachia* bacteria are common endosymbionts of insects, and some strains are known to protect their hosts against RNA viruses and other parasites. This has led to the suggestion that releasing *Wolbachia-*infected mosquitoes could prevent the transmission of arboviruses and other human parasites. We have identified *Wolbachia* in Kenyan populations of the yellow fever vector *Aedes bromeliae* and its relative *Aedes metallicus,* and in *Mansonia uniformis* and *Mansonia africana,* which are vectors of lymphatic filariasis. These *Wolbachia* strains cluster together on the bacterial phylogeny, and belong to bacterial clades that have recombined with other unrelated strains. These new *Wolbachia* strains may be affecting disease transmission rates of infected mosquito species, and could be transferred into other mosquito vectors as part of control programs.

## Introduction

*Wolbachia* are common intracellular bacteria that are estimated to infect 40% of insect species [1]. They are typically vertically transmitted from infected females to their offspring, and are best known for manipulating host reproduction in ways that enhance their own transmission to future host generations. The commonest manipulations involve either distorting the offspring sex ratio of infected females towards daughters [2]–[4] or inducing cytoplasmic incompatibility [5], [6]. These manipulations allow the bacteria to rapidly spread through insect populations, often to the point where virtually all individuals are infected [7].

Some *Wolbachia* strains also protect infected insects against RNA viruses, and have the potential to prevent the transmission of human pathogens by insect vectors [8]–[10]. This effect was first reported in *Drosophila,* where *Wolbachia-*infected insects had increased survival and/or lower viral titres when infected with a range of positive-sense RNA viruses [8], [10], [11]. Subsequently, it was found that when *D. melanogaster Wolbachia* strains were transinfected into *Ae. aegypti* or *Ae. albopictus,* the mosquitoes had increased resistance to dengue and chikungunya viruses [9], [12], [13]. Furthermore, the bacteria also induced cytoplasmic incompatibility in their new hosts, so they have the potential to spread through wild and naïve mosquito populations [12]–[14]. Therefore, releasing *Wolbachia-*infected mosquitoes may provide a way to interrupt the transmission cycle of these viruses.

The effects of *Wolbachia* on metazoan parasites of public health importance have also been investigated. A virulent strain of *Wolbachia*, *w*MelPop, which over-replicates in somatic tissues and reduces the lifespan of infected hosts [15], [16], caused an upregulation of immune genes responsive to filarial worm infections when transinfected into *Ae. aegypti* [17]. *w*MelPop also reduced the intensity of the avian malaria parasite, *Plasmodium gallinaceum*, in *Ae. aegypti* [9] and the rodent parasite, *P. berghei*, in *Anopheles gambiae* [18].

While the ability of *Wolbachia* to spread rapidly through populations and impair the development of pathogens makes it an excellent candidate for reducing disease transmission by vector species, the choice of *Wolbachia* strain for such interventions needs to be carefully considered. For example, while *w*MelPop can prevent the normal replication of viruses and development of metazoans, it also shortens the life of infected mosquitoes and this high fitness cost may prevent it from stably establishing in mosquito populations [16], [18]. Furthermore, *Wolbachia* strains vary considerably in both the level of viral protection that they provide to their hosts [11] and the strength of cytoplasmic incompatibility that they induce [19], [20]. These, in turn, affect the ability of the bacteria to spread through populations and prevent disease transmission. For these reasons it is prudent to consider a range of strains before beginning any control programs.

Identifying naturally-occurring *Wolbachia* strains in mosquitoes is useful for two reasons. First, it is technically easier to transfer *Wolbachia* between closely related species [21], so strains which naturally occur in mosquitoes are well-suited for transinfection into related vector species. Second, the dynamics of *Wolbachia* strains that are introduced into an insect population may be altered by *Wolbachia* strains that already exist in that population due to cytoplasmic incompatibility among strains [22]. It is therefore important to critically assess the incidence of *Wolbachia* in natural populations of mosquitoes in order to better understand how introducing novel *Wolbachia* strains may alter a wild population that already harbours the bacteria. Naturally-occurring *Wolbachia* have been identified in a range of species of mosquitoes [23]–[25], many of which are not vectors of human disease. Here, we continue these efforts and examine *Wolbachia* infections in nine different species mosquitoes collected from Kenya and use multi-locus sequence typing (MLST) [26] to investigate relationships and patterns of genetic exchange among strains.

## Methods

### Mosquito Samples and *Wolbachia* Infection

We used DNA extractions from the guts of adult female mosquitoes collected from towns and villages along the Kenyan coast that have been described elsewhere [27]. *Anopheles* and *Mansonia* mosquitoes were collected from rural Mbogolo in the Malindi district and, *Aedes* and *Culex* from towns in the Kilifi district. The analysis was repeated on the heads and thoraces of a subset of these samples, but this did not lead to the discovery of any new infections, so the results are not reported. To detect *Wolbachia,* the gene encoding the surface protein of *Wolbachia*, *wsp*, was amplified with the primers *wsp*81F and *wsp* 691R [28]. PCR was performed with BIOTAQ polymerase (Bioline, UK) with each reaction at a final volume of 20 µl (2 µl of 10X PCR buffer, 1 µl of 50 mM MgCl2, 2 µl of 2 mM dNTP mix (Invitrogen), 0.2 µl each of 20 µM forward and reverse primers, 1 U Taq polymerase, 1 µL DNA sample and deionized water to the final voulme). The thermal cycling protocol was an initial denaturation at 95°C for 5 mins; 30 cycles of denaturation at 95°C for 30 s, annealing at 55°C for 20 s and extension at 72°C for 20 s; final extension at 72°C for 10 mins and held at 4°C. To check the DNA extraction had been successful, the insect ribosomal internal transcribed spacer region-1 (ITS1) and mtDNA cytochrome oxidase I (COI) were amplified for each sample using BD1 and 4S primers [29] and universal COI primers [30] respectively, in separate reactions.

### *wsp* and ITS1 Sequencing

For each mosquito species that was infected with *Wolbachia*, a maximum of 4 positive samples were selected for sequencing both *wsp* and ITS1. Unincorporated primers and dNTPs were digested with exonuclease I (ExoI) (NEB) and shrimp alkaline phosphatase (USB Corporation). Cleaned products were sequenced with the forward and reverse primers for each amplicon using ABI PRISM BigDye Terminator kit (Perkin-Elmer Corporation, U.S.A). Sequencing was done at the Source Bioscience Center, UK. Sequences were trimmed and assembled using Sequencher v4.5 [31]. Chromatograms were inspected for single and double peaks.

### Multi Locus Sequence Typing (MLST)

For typing the *Wolbachia* strains detected in our infected samples, we used the multi-locus typing as described [26]. The protocol suggests the amplification of 5 single copy genes that are widely distributed within the *w*Mel genome – *gatB, coxA, hcpA, ftsZ* and *fbpA*. We selected two individuals from each of the mosquito species that were infected with *Wolbachia,* except for *Ae. metallicus* which had only one infected individual. We used a nested PCR to amplify *hcpA* for *M. uniformis* samples as these failed to amplify with the *hcpA* standard primers F1/R1. Firstly, *hcpA* F3/R3 primer set was used in a reaction with Promega GoTaq Hot Start Polymerase. Then, 1 µl of the F3/R3 reaction was used in the next round of PCR using the *hcpA* F1/R1 primers as already described. The PCR resulted in multiple bands for *M. uniformis*. The correct band size was excised and purified with Qiagen Gel extraction kit. All amplicons were cleaned and prepared for sequencing as previously described.

Forward and reverse sequences from each PCR product were aligned and visually inspected in Sequencher v4.5 [31]. Consensus sequences obtained from each individual for each gene were aligned and compared and, all sequence differences between *Wolbachia* strains were checked to confirm they had unambiguous peaks. As bacteria from each mosquito species had the same sequences, a consensus sequence for each gene per mosquito host species was obtained (except *fbpA* gene which was sequenced for one of the two *M. uniformis* samples). All consensus sequences were trimmed to the appropriate length for database query. We performed a BLAST search of each sequence in the *Wolbachia* MLST database (http://pubmlst.org/wolbachia) [32]. Where a sequence had an exact match in the database, it was assigned the designated allele number. We submitted 6 new alleles to the database for allele number assignment which includes all the genes for the *Aedes* sp. and *hcpA* for *M. africana*. The complete MLST profiles were submitted to the *Wolbachia* MLST database and have been assigned ID numbers 496–501 (http://pubmlst.org/wolbachia).

### Phylogeny

To account for the effects of recombination on the phylogeny, we analysed the dataset with ClonalFrame v1.2 [33]. Unlike other phylogeny analysis software, ClonalFrame estimates clonal relationships while taking into account recombination as a mode of substitution within genes. This approach also estimates the contribution made by recombination to total substitutions [33]. The complete dataset included *Wolbachia* MLST sequences from 113 host strains obtained from MLST database (http://pubmlst.org/wolbachia), and our 5 sample species. We also performed a whole-genome shotgun (wgs) BLAST search of the *w*AlbB genome [34] for the MLST gene sequences of the *Wolbachia* B strain of *Ae. albopictus*. For the data downloaded from http://pubmlst.org/wolbachia, only host strains with complete information – details on the host genus and allele numbers for all 5 MLST genes – were included. If there were multiple MLST profiles from a single host that had identical sequences, only one of these was included.

All 5 gene sequences for the 119 sample set were aligned independently using Mauve v2.3.1 [35], which produces the appropriate file format for running ClonalFrame [33]. To check the analysis was converging, we performed 9 independent runs of our dataset in ClonalFrame v1.2 [33] with 100,000 MCMC iterations after 100,000 burn-in iterations. The number of iterations performed between recording parameters in the posterior sample was set at 100. Default settings were used for all other parameters. For the first 8 runs we used a uniformly chosen coalescent tree as the initial tree. As a UPGMA gives a good representation of tree topology [33], we performed the ninth run with parameters as previously mentioned, but starting with a UPGMA tree.

The output generated with the UPGMA starting tree was compared with the other 8 ClonalFrame outputs using the tree comparison tool in ClonalFrame [33]. This tool compares nodes of an uploaded tree to other tree outputs and plots the nodes according to similarity in observed nodes. The UPGMA tree showed good convergence with the other outputs (Figure S1). Good convergence of the various parameter estimates was demonstrated by the Gelman and Rubin test [36] implemented in ClonalFrame [33]. The UPGMA starting tree output was, therefore, used in further analyses. The posterior sample of trees was exported into MEGA 5.05 [37] and a consensus tree with branch support values was drawn at 50% majority rule. The resulting tree was visualized and rooted in FigTree v1.3.1 [38].

Where recombination was detected in our sequences by ClonalFrame, we attempted to identify the source of the ‘imported’ sequence. We used the criteria used by [39] to detect sources of imports in the mosquito clades. We checked the MLST database for sequences with two or fewer nucleotide differences from the gene being investigated (by first finding sequences that clustered together on a neighbour joining tree and then manually inspecting these sequences). If this sequence came from a bacterium that did not cluster with our sample on the MLST tree, then we classed it as a potential source of the sequence ‘imported’ by recombination [39].

## Results

### *Wolbachia* Infections

We investigated the presence of *Wolbachia* in mosquito guts of nine mosquito species. Due to variation in *Wolbachia* tissue tropism, some strains may go undetected. Nevertheless, it can provide a minimum estimate of the prevalence of *Wolbachia*. Five of the species sampled were infected (Table 1). Consistent with previous studies, none of the *Anopheles* species or *Aedes aegypti* were infected with *Wolbachia* [24], [25], while *Culex quinquefasciatus* and *Mansonia uniformis* were infected [24]. However, this is the first report of *Wolbachia* in *Aedes bromeliae*, *Aedes metallicus* and *Mansonia africana*. In *Ae. bromeliae,* 75% of individuals were infected, while in the closely related *Ae. metallicus,* one of the two samples was infected. Differences in the length and sequence of the mosquito internal transcribed spacer region-1 (ITS1) confirmed that the infected *Ae. metallicus* individual was of a distinct species to *Ae. bromeliae*. For each mosquito species, there were no nucleotide polymorphisms in the *wsp* sequences and the chromatograms showed clear single peaks, suggesting that a single strain was infecting these mosquitoes.

**Table 1:** Prevalence of *Wolbachia* in mosquitoes from Kenya.

Species	Number individuals	*wsp* positive	Prevalence (%)
*Anopheles gambiae*	22	0	
*Anopheles funestus*	27	0	
*Anopheles coustani*	4	0	
*Culex quinquefasciatus*	24	10	42 (22–63)
*Mansonia uniformis*	19	5	26 (9–51)
*Mansonia africana*	22	6	27 (11–50)
*Aedes aegypti*	29	0	
*Aedes bromeliae*	16	12	75 (48–96)
*Aedes metallicus*	2	1	50 (1–99)

The prevalence is shown with the 95% confidence interval in parentheses.

### Phylogeny

**Figure 1:**
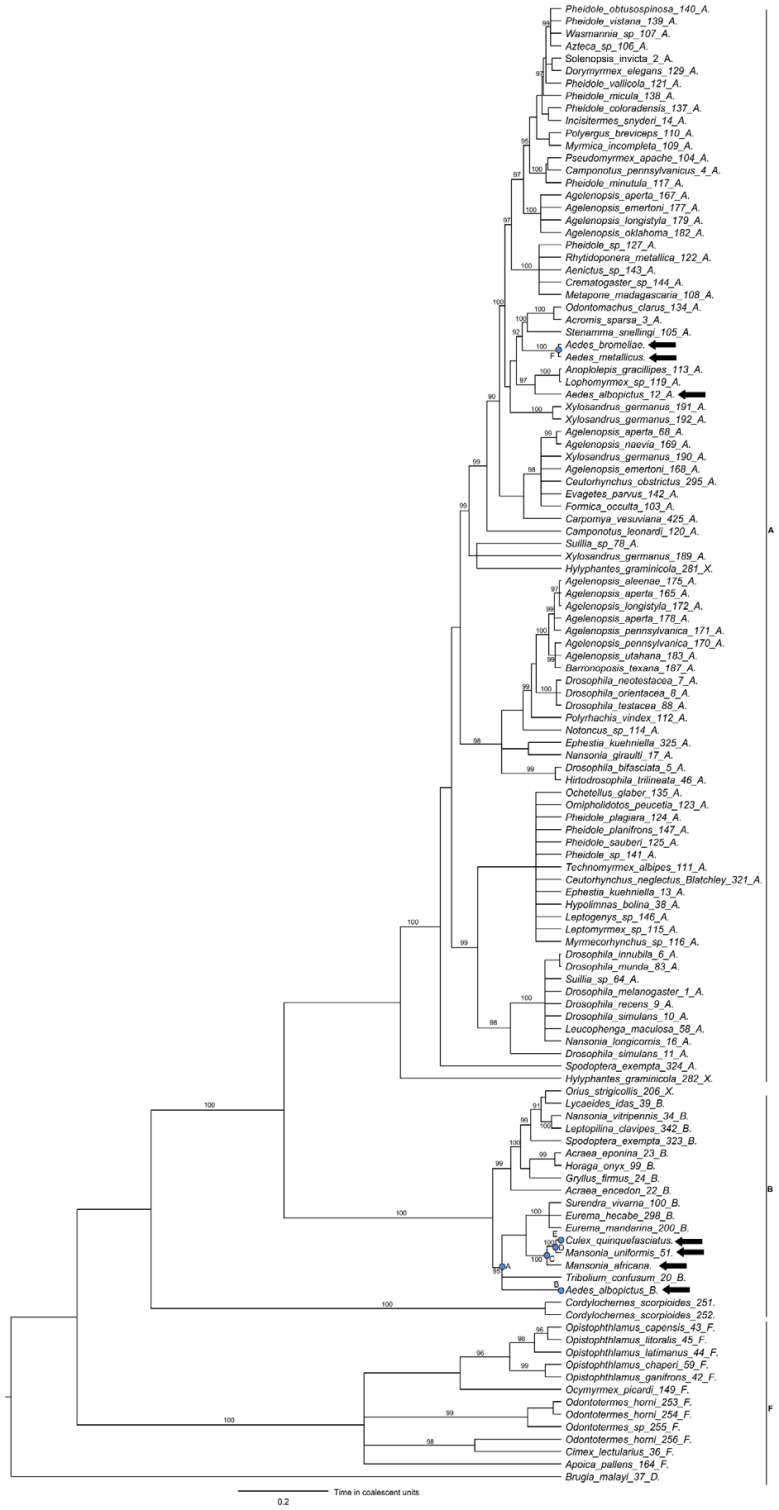
Phylogeny of *Wolbachia* strains based on *Wolbachia* MLST genes. Tip labels include 115 host strains from PubMLST (http://pubmlst/wolbachia) and 5 mosquito host species from this study. Black arrows indicate positions of mosquito *Wolbachia* strains. The branch labels are posterior probabilities (only support values above 90% are shown). Blue circles are the nodes leading to *Wolbachia* strains in mosquitoes that are analysed in Figure 2. Branch lengths are proportional to divergence time in coalescent units. The tree was constructed using the Bayesian ClonalFrame software.

We used the MLST gene sequences to reconstruct the *Wolbachia* phylogeny, and controlled for the confounding effects of recombination on tree reconstruction using ClonalFrame (Figure 1). The phylogeny grouped strains into supergroups A, B, F and D (Figure 1; supergroup D was used as the outgroup to root the tree) [40]–[43]. The *Wolbachia* strains we identified infecting the Culicini and Mansoniini tribes of mosquitoes belonged to supergroup B while those in the Aedini tribe were in supergroup A (Figure 1). *Wolbachia* strains in *Ae. bromeliae* and *Ae. metallicus* formed a monophyletic group, whose relationship with the strain from *Ae. albopictus Wolbachia* strain A (Figure 1; Aedes_albopictus_12_A) is poorly resolved. The three strains that infect *Culex quinquefasciatus, Mansonia uniformis* and *Mansonia africana* clustered together with three strains found in the Lepitoptera (Figure 1). There are numerous other strains from this clade in the MLST database, many of which infect Lepidoptera, and if these are included in the tree the relationships within the clade are poorly resolved (data not shown).

### Recombination Events

Across the entire tree of 119 strains, we estimated that recombination involves a mean tract length of 127 bp being exchanged between strains (95% credibility interval: 98–164 bp). We estimated that recombination (*r*) and mutation (*m*) had a similar probability of introducing substitutions into the genome of *Wolbachia* (mean *r/m* = 1.36; 95% credibility interval: 1.01–1.79). Although both events may have equal chances of producing nucleotide substitutions, the rate at which each occurs could be different. Defined by ρ/θ (recombinational to mutational rate), point mutations were estimated to happen roughly four times more frequently than recombination (ρ/θ = 0.27, 95% credibility interval: 0.19–0.37).

**Figure 2:**
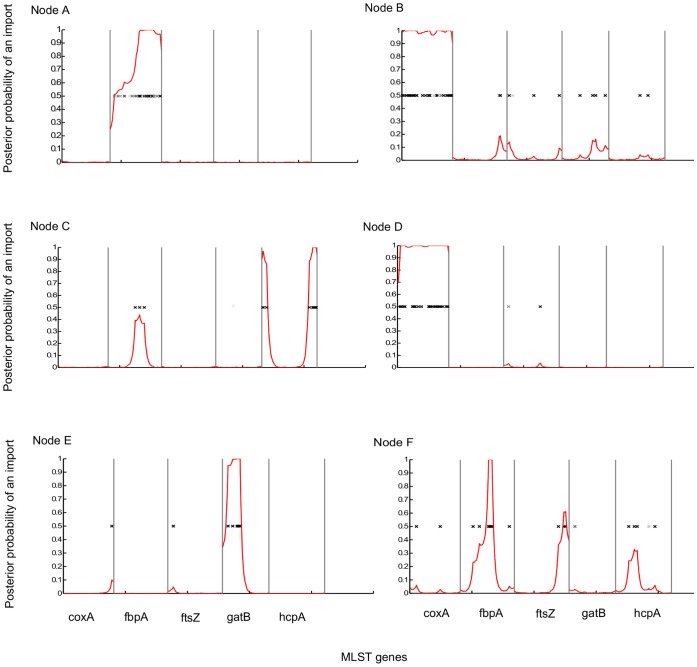
Recombination events on branches leading to nodes in the mosquito *Wolbachia* clades. The nodes A–F are shown by blue circles in Figure 1. The red line is åthe posterior probability that the sequence was imported by recombination. Positions marked ‘x’ are nucleotide substitutions in the genes; intensity of ‘x’ markings are proportional to posterior probability of substitution with darker marks indicating higher probability.

We were specifically interested in recombination that involved *Wolbachia* strains related to those infecting mosquitoes. We inspected the substitutions that had occurred on individual branches leading to various nodes in the mosquito clades (shown by blue circles in Figure 1). There were several lineages where there was a high probability of recombination (posterior probability of import >0.95; Figure 2). On the branch that leads to the mosquito *Wolbachia* clade in supergroup B (Figure 2; node A), about a third of the length of the *fbpA* gene was imported. In the *Aedes albopictus Wolbachia* strain B (*w*AlbB) the full length of the *coxA* gene was imported (Figure 2; node B), differentiating this strain from those of the other mosquito species in this supergroup. Similarly, the entire *coxA* gene was imported on the lineage leading to the strains infecting *Mansonia uniformis* and *Culex quinquefasciatus* (Figure 2; node D). There was also evidence of three smaller recombination events in the mosquito clades (Figure 2; nodes C, E and F).

Since the *coxA* gene has a high probability of the entire gene having been exchanged by recombination (Figure 2; nodes B and D), we investigated the possible sources of these imported sequences. To do this, we reconstructed a neighbour-joining tree using all published sequences of *coxA*, and looked for taxa which had very similar sequences (two or fewer differences) to the sequences from the *Wolbachia* strains that showed evidence of recombination but appeared elsewhere on the MLST tree. The source of *coxA* into *Ae. albopictus w*AlbB was not detected in the database. The source of the *coxA* sequence into the lineage on node D (Figure 2) appears to be host strains 355, 502, 439 and 492 in the MLST database (data not shown). Unfortunately, the names of the arthropod species that these strains infect have not been published.

## Discussion

*Wolbachia* is an important component of the antiviral defences of insects [8], [10], [11], which has the potential to prevent mosquitoes from transmitting viruses like dengue and chikungunya [9], [13]. Furthermore, some strains of *Wolbachia* also affect metazoan parasites like *Plasmodium* [9], hence they may also play a role in affecting the transmission of these parasites [17], [18]. Here, we make the first report of *Wolbachia* infections in *Ae. bromeliae*, a vector of yellow fever virus [44], and *M. africana,* a vector of the filarial nematode *Wuchereria bancrofti* [45] – a major cause of lymphatic filariasis. Furthermore, we extend the known range of *Wolbachia* in *M. uniformis* from Southeast Asia [24] to Africa, where this species is a competent vector of *Wuchereria bancrofti* [45].

The *Wolbachia* strains we have identified may have implications for both the natural transmission rate of human disease, and the attempts to manipulate transmission rates through the release of *Wolbachia*-infected mosquitoes. As virus protection appears to be a common trait among *Wolbachia* strains in arboviral hosts [8], [10], [11], it is possible that these strains we have detected in the yellow fever vector *Ae. bromeliae* may reduce arboviral transmission rates in the wild. This has the potential to be significantly important since 75% of individuals were infected. These strains also have the potential to be transinfected into key vector species such as *Ae. aegypti.* This is likely to be far easier than transfers of strains from distantly related species like *Drosophila,* as transinfection is known to have higher success rates between more closely related species of insects [21], [46]. Finally, these resident *Wolbachia* strains might interfere with attempts to introduce novel strains into the population as part of control programs.

*Wolbachia* bacteria were first reported in *C. pipiens* [47] and since then many more strains have been reported in other mosquito genera [24], [25], [48], [49]. Our sample size was not as large and diverse as previous work have shown [24], [49] but, the presence of *Wolbachia* in the genera *Aedes*, *Culex* and *Mansonia* is confirmed, and the prevalence of infections in the three genera of mosquitoes was comparable to other studies [24]. The failure to detect *Wolbachia* in the three species of *Anopheles* in our study confirms the absence of *Wolbachia* in this group of mosquitoes [24], [49]. It was speculated that this may be due to the inability of Anophelines to support *Wolbachia* physiologically [24]. Recently, transinfection of *Wolbachia* into *Anopheles* has shown stable infections in somatic cells with striking effects on immune gene regulation in response to *Plasmodium falciparum* but, no infections in the ovaries was observed [50], [51].

With the confounding effects of recombination accounted for, we have provided analyses of the *Wolbachia* phylogeny. The *Wolbachia* strains in mosquitoes were clearly categorized into supergroups A and B, with at least two mosquito-infecting clades. The tendency of the mosquito-infecting strains to cluster together could be explained by an ancestral species being infected and then co-speciating with *Wolbachia,* or by horizontal transmission between mosquitoes. Horizontal transfer seems more probable, as it is known from other taxa that horizontal transmission most commonly occurs between the closest related host species [52].

## Supporting Information

Figure S1 **ClonalFrame ‘tree comparison’ tool output of UPGMA starting tree with the 8 coalescent tree models.** Shaded circles are proportional to the support on the nodes during the tree comparison. Black circles represent nodes that are present in the UPGMA starting trees and all the other trees, white nodes are nodes found on the UPGMA starting tree but not in the combined comparison with the other tree outputs.
(PDF)
