# Supplementary material for: Identification of Wolbachia Strains in Mosquito Disease Vectors
Source: PLoS One. 2012 Nov 21;7(11):e49922. doi: 10.1371/journal.pone.0049922 (PMC3503815; doi:10.1371/journal.pone.0049922)
Supplement: Figure S1 — ClonalFrame ‘tree comparison’ tool output of UPGMA starting tree with the 8 coalescent tree models. Shaded circles are proportional to the support on the nodes during the tree comparison. Black circles represent nodes that are present in the UPGMA starting trees and all the other trees, white nodes are nodes found on the UPGMA starting tree but not in the combined comparison with the other tree outputs. (PDF) [file pone.0049922.s001.pdf]

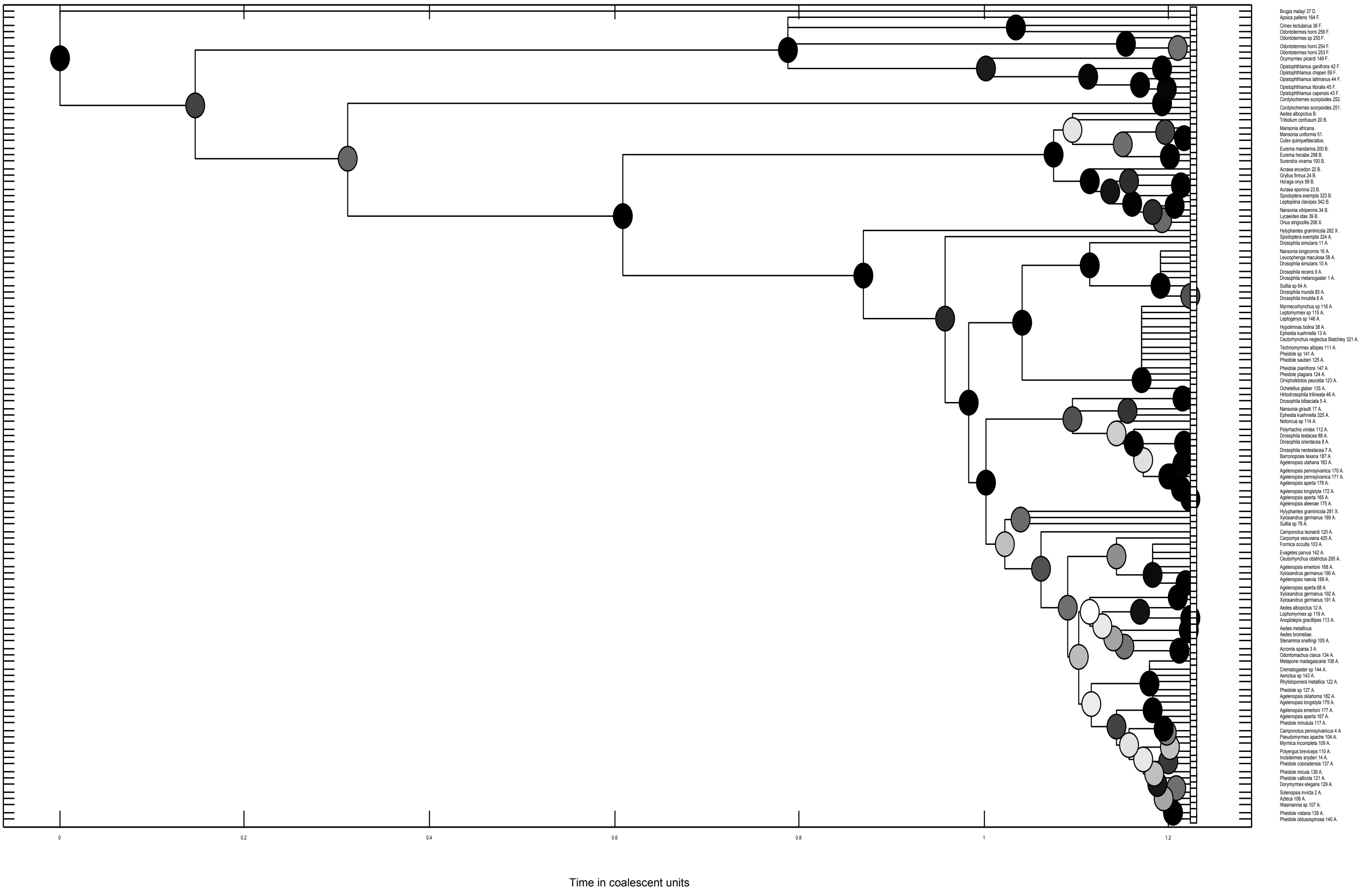
Brugia malayi 37 D.
Apoica pallens 164 F.
Cimex lectularius 36 F.
Odontotermes horni 256 F.
Odontotermes sp 255 F.
Odontotermes horni 254 F.
Odontotermes horni 253 F.
Ocymyrmex picardi 149 F.
Opistophthlamus ganifrons 42 F.
Opistophthlamus chaperi 59 F.
Opistophthlamus latimanus 44 F.
Opistophthlamus litoralis 45 F.
Opistophthlamus capensis 43 F.
Cordylochernes scorpioides 252.
Cordylochernes scorpioides 251.
Aedes albopictus B.
Tribolium confusum 20 B.
Mansonia africana.
Mansonia uniformis 51.
Culex quinquefasciatus.
Eurema mandarina 200 B.
Eurema hecabe 298 B.
Surendra vivarna 100 B.
Acraea encedon 22 B.
Gryllus firmus 24 B.
Horaga onyx 99 B.
Acraea eponina 23 B.
Spodoptera exempta 323 B.
Leptopilina clavipes 342 B.
Nansonia vitripennis 34 B.
Lycaeides idas 39 B.
Orius strigicollis 206 X.
Hylyphantes graminicola 282 X.
Spodoptera exempta 324 A.
Drosophila simulans 11 A.
Nansonia longicornis 16 A.
Leucophenga maculosa 58 A.
Drosophila simulans 10 A.
Drosophila recens 9 A.
Drosophila melanogaster 1 A.
Suillia sp 64 A.
Drosophila munda 83 A.
Drosophila innubila 6 A.
Myrmecorhynchus sp 116 A.
Leptomyrmex sp 115 A.
Leptogenys sp 146 A.
Hypolimnas bolina 38 A.
Ephestia kuehniella 13 A.
Ceutorhynchus neglectus Blatchley 321 A.
Technomyrmex albipes 111 A.
Pheidole sp 141 A.
Pheidole sauberi 125 A.
Pheidole planifrons 147 A.
Pheidole plagiara 124 A.
Ornipholidotos peucetia 123 A.
Ochetellus glaber 135 A.
Hirtodrosophila trilineata 46 A.
Drosophila bifasciata 5 A.
Nansonia giraulti 17 A.
Ephestia kuehniella 325 A.
Notoncus sp 114 A.
Polyrhachis vindex 112 A.
Drosophila testacea 88 A.
Drosophila orientacea 8 A.
Drosophila neotestacea 7 A.
Barronoposis texana 187 A.
Agelenopsis utahana 183 A.
Agelenopsis pennsylvanica 170 A.
Agelenopsis pennsylvanica 171 A.
Agelenopsis aperta 178 A.
Agelenopsis longistyla 172 A.
Agelenopsis aperta 165 A.
Agelenopsis aleenae 175 A.
Hylyphantes graminicola 281 X.
Xylosandrus germanus 189 A.
Suillia sp 78 A.
Camponotus leonardi 120 A.
Carpomya vesuviana 425 A.
Formica occulta 103 A.
Evagetes parvus 142 A.
Ceutorhynchus obstrictus 295 A.
Agelenopsis emertoni 168 A.
Xylosandrus germanus 190 A.
Agelenopsis naevia 169 A.
Agelenopsis aperta 68 A.
Xylosandrus germanus 192 A.
Xylosandrus germanus 191 A.
Aedes albopictus 12 A.
Lophomyrmex sp 119 A.
Anoplolepis gracilipes 113 A.
Aedes metallicus.
Aedes bromeliae.
Stenamma snellingi 105 A.
Acromis sparsa 3 A.
Odontomachus clarus 134 A.
Metapone madagascaria 108 A.
Crematogaster sp 144 A.
Aenictus sp 143 A.
Rhytidoponera metallica 122 A.
Pheidole sp 127 A.
Agelenopsis oklahoma 182 A.
Agelenopsis longistyla 179 A.
Agelenopsis emertoni 177 A.
Agelenopsis aperta 167 A.
Pheidole minutula 117 A.
Camponotus pennsylvanicus 4 A.
Pseudomyrmex apache 104 A.
Myrmica incompleta 109 A.
Polyergus breviceps 110 A.
Incisitermes snyderi 14 A.
Pheidole coloradensis 137 A.
Pheidole micula 138 A.
Pheidole vallicola 121 A.
Dorymyrmex elegans 129 A.
Solenopsis invicta 2 A.
Azteca 106 A.
Wasmannia sp 107 A.
Pheidole vistana 139 A.
Pheidole obtusospinosa 140 A.
0
0.2
0.4
0.6
0.8
1
1.2
Time in coalescent units
